# Insulin-treated diabetes is not associated with increased mortality in critically ill patients

**DOI:** 10.1186/cc8866

**Published:** 2010-02-04

**Authors:** Jean-Louis Vincent, Jean-Charles Preiser, Charles L Sprung, Rui Moreno, Yasser Sakr

**Affiliations:** 1 Department of Intensive Care, Erasme Hospital, Université libre de Bruxelles, route de Lennik 808, 1070 Bruxelles, Belgium; 2 Department of General Intensive Care, University Hospital Centre of Liege, Domaine Universitaire du Sart Tilman B 35, 4000 Liege, Belgium; 3 Department of Anesthesiology and Critical Care Medicine, Hadassah Hebrew University Medical Center, P.O.B. 12000, 91120 Jerusalem, Israel; 4 Department of Intensive Care, Hospital de St Antonio dos Capuchos, Alameda de Santo António dos Capuchos, 1169-050 Lisbon, Portugal; 5 Department of Anesthesiology and Intensive Care, Friedrich-Schiller-University, Erlanger Allee 101, Jena 07743, Germany

## Abstract

**Introduction:**

This was a planned substudy from the European observational Sepsis Occurrence in Acutely ill Patients (SOAP) study to investigate the possible impact of insulin-treated diabetes on morbidity and mortality in ICU patients.

**Methods:**

The SOAP study was a cohort, multicenter, observational study which included data from all adult patients admitted to one of 198 participating ICUs from 24 European countries during the study period. For this substudy, patients were classified according to whether or not they had a known diagnosis of insulin-treated diabetes mellitus. Outcome measures included the degree of organ dysfunction/failure as assessed by the sequential organ failure assessment (SOFA) score, the occurrence of sepsis syndromes and organ failure in the ICU, hospital and ICU length of stay, and all cause hospital and ICU mortality.

**Results:**

Of the 3147 patients included in the SOAP study, 226 (7.2%) had previously diagnosed insulin-treated diabetes mellitus. On admission, patients with insulin-treated diabetes were older, sicker, as reflected by higher simplified acute physiology system II (SAPS II) and SOFA scores, and more likely to be receiving hemodialysis than the other patients. During the ICU stay, more patients with insulin-treated diabetes required renal replacement therapy (hemodialysis or hemofiltration) than other patients. There were no significant differences in ICU or hospital lengths of stay or in ICU or hospital mortality between patients with or without insulin-treated diabetes. Using a Cox proportional hazards regression analysis with hospital mortality censored at 28-days as the dependent factor, insulin-treated diabetes was not an independent predictor of mortality.

**Conclusions:**

Even though patients with a history of insulin-treated diabetes are more severely ill and more likely to have renal failure, insulin-treated diabetes is not associated with increased mortality in ICU patients.

## Introduction

Diabetes mellitus is an increasingly common condition, and is estimated to affect approximately 246 million adults worldwide [1]. Although diabetes is occasionally the reason for admission to an intensive care unit (ICU), it is more commonly present as a comorbid condition. Although hyperglycemia can induce a number of immunological alterations [2-5], whether patients with diabetes who are admitted to the ICU are more likely to develop infectious complications remains a controversial issue with studies yielding conflicting results [6-12]. Similarly, some studies [11,13,14], but not all [10,15], have indicated increased mortality in ICU patients with diabetes.

In view of the relative lack of data on patients in the ICU with diabetes and the conflicting results from the available data, we investigated the potential impact of insulin-treated diabetes on morbidity and mortality in ICU patients included in a large European epidemiological study, the Sepsis Occurrence in Acutely ill Patients (SOAP) study [16].

## Materials and methods

The SOAP study was a prospective, multicenter, observational study designed to evaluate the epidemiology of sepsis, as well as other characteristics, of ICU patients in European countries. Details of recruitment, data collection, and management have been published previously [16]. Briefly, all patients older than 15 years admitted to the 198 participating centers [see the list of participating countries and centers in Additional data file 1] between 1 and 15 May, 2002, were included, except patients who stayed in the ICU for less than 24 hours for routine postoperative observation. Patients were followed until death, hospital discharge, or for 60 days. Due to the observational nature of the study, institutional review board approval was either waived or expedited in participating institutions and informed consent was not required.

Data were collected prospectively using pre-printed case report forms. Data collection on admission included demographic data and comorbidities, including diabetes requiring insulin administration. Clinical and laboratory data for the simplified acute physiology score (SAPS) II [17] were reported as the worst value within 24 hours after admission. Microbiologic and clinical infections were reported daily as well as the antibiotics administered. A daily evaluation of organ function according to the sequential organ failure assessment (SOFA) score [18], was performed, with the most abnormal value for each of the six organ systems (respiratory, renal, cardiovascular, hepatic, coagulation, and neurological) collected on admission and every 24 hours thereafter. Infection was defined as the presence of a pathogenic microorganism in a sterile milieu (such as blood, abscess fluid, cerebrospinal fluid or ascitic fluid), and/or clinically documented infection, plus the administration of antibiotics. Sepsis was defined according to consensus conference definitions as infection plus two systemic inflammatory response syndrome (SIRS) criteria [19]. Organ failure was defined as a SOFA score above two for the organ in question [20]. Severe sepsis was defined as sepsis with at least one organ failure.

For the purposes of this study, patients were separated into two groups according to whether or not they had a history of insulin-treated diabetes prior to ICU admission. The *a priori *defined outcome parameters for this analysis included the degree of organ dysfunction/failure as assessed by the SOFA score, the occurrence of sepsis syndromes and organ failure in the ICU, hospital and ICU lengths of stay, and all-cause hospital and ICU mortality.

### Statistical methods

Data were analyzed using SPSS 13.0 for Windows (SPSS Inc., Chicago, IL, USA). Descriptive statistics were computed for all study variables. A Kolmogorov-Smirnov test was used, and histograms and normal-quantile plots were examined to verify the normality of distribution of continuous variables. Discrete variables are expressed as counts (percentage) and continuous variables as means ± standard deviation or median (25th to 75th percentiles). For demographic and clinical characteristics of the study groups, differences between groups were assessed using a chi-squared, Fisher's exact test, Student's t-test or Mann-Whitney U test, as appropriate.

We performed a Cox proportional hazards regression analysis to examine whether the presence of diabetes was associated with mortality. To correct for differences in patient characteristics, we simultaneously included age, gender, SAPS II score on admission, co-morbidities, type of admission (medical or surgical), infection on admission, mechanical ventilation on admission, renal replacement therapy on admission (hemofiltration or hemodialysis), renal failure on admission, and creatinine level on admission. Variables were introduced in the model if significantly associated with a higher risk of 28-day in-hospital death on a univariate basis at a *P *value less than 0.2. Colinearity between variables was excluded prior to modelling. Extended Cox models were constructed adding interaction terms. The most parsimonious model was fitted and retained as the final model. We tested the assumption of proportionality of hazards and found no evidence of violation. We also tested the qualitative goodness of fit of the model. All statistics were two-tailed and a *P *less than 0.05 was considered to be statistically significant.

## Results

Of the 3147 patients included in the SOAP study, 226 (7.2%) had a prior diagnosis of insulin-treated diabetes mellitus. Table 1 presents the characteristics of the study group on admission to the ICU. Patients with a history of insulin-treated diabetes were older (66 (range 55 to 75) versus 64 (49 to 74) years, *P *< 0.01) and more severely ill on admission, as reflected by the higher SAPS II and SOFA scores, than were patients without a history of insulin-treated diabetes. On admission, more patients with a history of insulin-treated diabetes had renal failure and were undergoing hemodialysis than did patients with no history of insulin-treated diabetes. On admission and during the ICU stay, there were no differences in the occurrence of sepsis or septic shock among ICU patients with and those without a history of insulin-treated diabetes (Tables 1 and 2). During the ICU stay, more patients with a history of insulin-treated diabetes developed renal failure and underwent hemodialysis than did those without a history of insulin-treated diabetes (Table 2).

**Table 1:** Characteristics of the study group on admission to the intensive care unit in patients with and without a history of insulin-treated diabetes.

	No history of insulin-treated diabetes (n = 2921)	History of insulin-treated diabetes (n = 226)	*P *value
Age, years, median (IQR)	64 (49-74)	66 (55-75)	< 0.01
Sex, male n (%)	1790 (62)	130 (58)	0.2
Medical admission, n (%)	1301 (45)	87 (39)	0.08
**Reason for admission**			
Digestive/liver	312 (11.3)	21 (10.1)	0.35
Respiratory	519 (18.8)	41 (19.7)	0.71
Cardiovascular	878 (31.8)	71 (34.1)	0.49
Hematological	26 (0.9)	1 (0.5)	0.99
Neurological	455 (16.5)	30 (14.4)	0.5
Renal	86 (3.1)	18 (8.7)	< 0.01
Metabolic	56 (2)	15 (7.2)	< 0.01
Trauma	178 (6.4)	3 (1.4)	< 0.01
**Comorbid conditions**			
Cancer, n (%)	390 (13)	25 (11)	0.36
Hematological cancer	67 (2.3)	2 (0.9)	0.34
COPD	317 (10.9)	23 (10.2)	0.82
HIV infection	24 (0.8)	2 (0.9)	0.84
Liver cirrhosis	110 (3.8)	11 (4.9)	0.37
Heart failure	259 (8.9)	48 (21.2)	< 0.001
**Presence of sepsis, n (%)**			
Sepsis	717 (25)	60 (27)	0.52
Severe sepsis	503 (17)	49 (22)	0.10
Septic shock	227 (7.8)	16 (7.1)	0.80
**Renal failure on admission**	519 (17.8)	56 (24.8)	0.01
With hemodialysis	27 (0.9)	10 (4.4)	< 0.001
Without hemodialysis	492 (16.8)	46 (20.4)	0.20
**Interventions, n (%)**			
Mechanical ventilation	1720 (59)	130 (58)	0.73
Hemofiltration	65 (2)	8 (4)	0.24
Hemodialysis	36 (1)	13 (6)	< 0.001
**Creatinine, mg/dL**	1.42 ± 1.40	1.93 ± 1.90	< 0.001
**SAPS II, median (IQR)**	34 (24-46)	36 (26-49)	0.02
**SOFA score, median (IQR)**	6 (4-9)	8 (4-10)	< 0.01

COPD = chronic obstructive pulmonary disease; IQR = interquartile range; SAPS = simplified acute physiology score; SOFA = sequential organ failure assessment.

**Table 2:** Procedures, organ failures, and presence of infection during the ICU stay, and ICU and hospital outcomes in patients with and without a history of insulin-treated diabetes

	No history of insulin-treated diabetes (n = 2921)	History of insulin-treated diabetes (n = 226)	*P *value
Infection, n (%)			
Before 48 hours	825 (28)	73 (32)	0.19
After 48 hours (ICU acquired)	263 (9)	16 (7)	0.33
Sepsis, n (%)	1088 (37)	89 (39)	0.52
Severe sepsis, n (%)	855 (29)	75 (33)	0.23
Septic shock, n (%)	423 (15)	39 (17)	0.28
Procedures, n (%)			
Mechanical ventilation, at least once	1886 (65)	139 (62)	0.35
Hemofiltration, at least once	187 (6)	24 (11)	0.02
Hemodialysis, at least once	111 (4)	30 (13)	< 0.001
Organ dysfunction (any time), n (%)			
Renal failure	1015 (35)	105 (47)	< 0.01
with hemodialysis on admission	32 (1.1)	11 (4.9)	< 0.001
without hemodialysis on admission	983 (34)	94 (42)	0.02
Respiratory failure	1202 (41)	99 (44)	0.44
Coagulation failure	289 (10)	20 (9)	0.73
Hepatic failure	154 (5.3)	14 (6)	0.54
CNS failure	782 (27)	57 (25)	0.64
Cardiovascular failure	971 (33)	81 (36)	0.42
Organ dysfunction (after 48 hours), n (%)			
Renal failure	248 (9)	23 (10)	0.38
Respiratory failure	208 (7)	18 (8)	0.64
Coagulation failure	75 (3)	6 (3)	0.73
Hepatic failure	51 (2)	4 (2)	0.98
CNS failure	76 (3)	5 (2)	0.72
Cardiovascular failure	93 (3)	10 (4)	0.31
ICU LOS, days, median (IQR)	3 (2-7)	3 (2-8)	0.49
Hospital stay, days, median (IQR)	15 (7-32)	17 (9-35)	0.15
ICU mortality, n (%)	540 (19)	43 (19)	0.86
Hospital mortality, n (%)	684 (24)	63 (28)	0.15

CNS = central nervous system; ICU = intensive care unit; IQR = interquartile range; LOS = length of stay.

**Figure 1:**
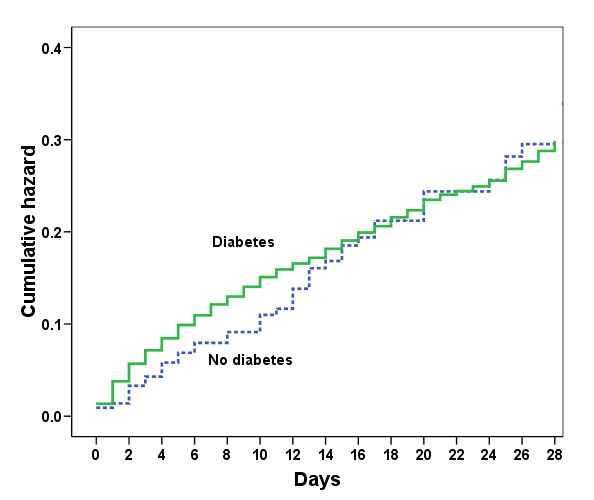
**Cumulative hazard of death during the first 28 days in the intensive care unit in patients with and without a history of insulin-treated diabetes**.

There were no differences in ICU or hospital lengths of stay in patients with or without a history of insulin-treated diabetes and ICU and hospital mortality rates were also similar (Table 2). In the Cox regression model, medical admission, higher SAPS II score, older age comorbid liver cirrhosis, and mechanical ventilation on admission, but not a history of insulin-treated diabetes, were associated with an increased risk of death at 28 days (Table 3 and Figure 1).

**Table 3:** Summary of Cox proportional hazards model analysis with time to hospital death right-censored at 28 days as the dependent factor.

	B	SE	HR	95% CI	P
Medical admission	0.71	0.094	2.04	1.70 - 2.45	< 0.001
Age, year	0.01	0.003	1.01	1.00 - 1.02	0.001
SAPS II score (per point)	0.04	0.002	1.05	1.04 - 1.05	< 0.001
Mechanical ventilation, on admission	0.30	0.111	1.35	1.09 - 1.68	0.007
Liver cirrhosis on admission	0.79	0.160	2.19	1.60 -- 3.00	< 0.001
Insulin-treated diabetes	-0.24	0.157	0.78	0.58 - 1.07	0.120

B = coefficient estimate; CI = confidence interval; HR = hazard ratio; SAPS = simplified acute physiology score; SE = standard error of the estimate.

## Discussion

The present results demonstrate that in this heterogeneous population of critically ill patients in Western Europe, patients with a history of insulin-treated diabetes had similar mortality rates to those without, even though patients with a history of insulin-treated diabetes were more severely ill on admission to the ICU and were more likely to have or to develop renal failure and to require hemodialysis than patients with no history of insulin-treated diabetes. Importantly, these results refer to patients who were receiving insulin on admission and do not reflect the effects of insulin treatment during the hospital stay. The development of renal failure in ICU patients is generally associated with an increase in mortality [21,22]; however, this was not the case in our patients, perhaps because in the majority of the patients renal failure was already present on admission, making it a less important prognostic factor than renal failure that develops later during the ICU admission.

Although diabetes is a relatively common comorbidity in critically ill patients - in our study 7% of patients had a history of insulin-treated diabetes - its effects on outcomes have not been extensively studied. In the literature, there seems to be considerable variation regarding the effect of diabetes on outcomes in different groups of critically ill patients. In an analysis of a database of 15,408 individuals, Slynkova and colleagues [14] reported that patients with a history of diabetes mellitus were three times more likely to develop acute organ failure and had a threefold risk of dying when hospitalized for that organ failure. In patients with community-acquired pneumonia, diabetes was an independent predictor of mortality in a multivariate analysis in one study [23], but it was not associated with increased mortality in patients with community-acquired bacteremia in another study [24]. In patients with acute myocardial infarction, diabetes has been associated with increased short-term [25] and long-term [26] mortality; however, in trauma patients, Ahmad and colleagues reported that although patients with diabetes had more complications and longer hospital stays, they did not have higher mortality rates than non-diabetic patients [10]. Also in trauma patients, Kao and colleagues reported that diabetes was associated with increased infectious complications but not with increased mortality [27]. Similar findings have been reported in burn patients [9] and in patients with acute heart failure [28]. In patients undergoing hepatic resection, patients with a history of diabetes had higher rates of postoperative renal failure, but diabetes was not an independent risk factor for mortality [29]. In patients with severe sepsis or septic shock enrolled in a large multicenter trial, Stegenga and colleagues recently reported that patients with a history of diabetes had similar 28-day and 90-day mortality rates to the other patients [30]. In the present study, the incidence of infections acquired during the ICU stay was not higher in patients with a history of insulin-treated diabetes; however, this does not exclude the possibility that some specific subgroups (e.g., cardiac surgery) of diabetic patients may more frequently experience postoperative infections as suggested in other studies [11].

Much has been written in recent years about the potential role of hyperglycemia on admission [31] and during the ICU stay [32,33] on outcomes in ICU patients and the need for tight control of glucose concentrations using insulin [34-38]. Hyperglycemia has been associated with impaired neutrophil chemotaxis, oxidative burst, and phagocytosis and increased neutrophil adherence [2-5]. Using intravital microscopy, Booth and colleagues demonstrated that hyperglycemia was able to initiate an inflammatory response in the microcirculation [39], and correction of hyperglycemia in critically ill patients has been associated with improved outcomes [34,40]. Our present study was not focused on hyperglycemia. Whether or not blood glucose should be strictly controlled is a different issue, which requires prospective, controlled, randomized studies as in the study by Van den Berghe and colleagues in which surgical ICU patients who were managed with a strict protocol to maintain blood glucose concentrations between 80 and 110 mg/dl (4.4 and 6.1 mmol/l) had less morbidity and lower mortality than patients treated conventionally [34]. This approach is still very controversial [35]. Interestingly, in these studies by Van den Berghe and colleagues [34,40], patients who had a history of diabetes did not benefit from the tight glucose control approach [36]. Several other studies have also indicated that, although many ICU patients with newly diagnosed or stress hyperglycemia have worse outcomes than normoglycemic patients, this relation does not hold true or is less marked for patients with known diabetes [41-46]. In the recent SAPS III study, diabetes, with or without insulin treatment, was associated with a worse hospital mortality in multivariate analysis [47]. Interestingly, diabetic patients with septic shock may have a lower incidence of developing acute lung injury or acute respiratory distress syndrome[48,49].

The present study has some limitations including that, as part of an observational study with a waiver of informed consent, we were unable to obtain glycosylated hemoglobin measurements and also did not have blood glucose levels to evaluate the degree of control of the diabetes before or during the ICU admission. In addition, we compared patients with a history of insulin-treated diabetes to a cohort consisting of non-diabetics and non-insulin-treated diabetics, and have no data on the numbers of non-insulin-treated diabetics in this cohort. More importantly, we did not separate patients with type 1 and type 2 diabetes because this information is difficult to define in ICU patients. The slightly higher proportion of medical patients in the non-diabetic group could represent a confounding factor, because mortality is usually higher in medical than in surgical ICU patients. Finally, we evaluated a heterogeneous patient population but the multivariate analysis we performed adjusted for a large number of variables, which are known to influence outcome prediction.

## Conclusions

In conclusion, in this general ICU population, although patients with a history of insulin-treated diabetes were more severely ill and more likely to have renal failure, insulin-treated diabetes was not associated with increased ICU or hospital mortality rates.

## Key messages

• Patients with a history of insulin-treated diabetes are more severely ill on admission to the ICU and more likely to have or develop renal failure and to require hemodialysis than patients with no history of insulin-treated diabetes.

• However, ICU and hospital mortality rates were similar in patients with and without a history of insulin-treated diabetes.

## Abbreviations

ICU: intensive care unit; SAPS: simplified acute physiology score; SIRS: systemic inflammatory response syndrome; SOAP: sepsis in acutely ill patients; SOFA: sequential organ failure assessment.

## Competing interests

The authors declare that they have no competing interests.

## Authors' contributions

JLV conceived the initial SOAP study. JCP, CLS, RM, YS, and JLV participated in the design and coordination of the SOAP study. YS performed the statistical analyses. YS and JLV drafted the present manuscript. All authors read and approved the final manuscript.

## Supplementary Material

Additional file 1 **SOAP participants**. A word file listing the participants in the Sepsis Occurrence in Acutely Ill Patients (SOAP) study in alphabetical order. Click here for file
